# A rapid review of the impact of COVID-19 on the mental health of healthcare workers: implications for supporting psychological well-being

**DOI:** 10.1186/s12889-020-10070-3

**Published:** 2021-01-09

**Authors:** Johannes H. De Kock, Helen Ann Latham, Stephen J. Leslie, Mark Grindle, Sarah-Anne Munoz, Liz Ellis, Rob Polson, Christopher M. O’Malley

**Affiliations:** 1 University of the Highlands and Islands, Institute for Health Research and Innovation, University of the Highlands and Islands, Old Perth Road, Inverness, IV2 3JH UK; 2 NHS Highland, Department of Clinical Psychology, New Craigs Hospital, Inverness, IV3 8NP UK; 3 NHS Highland, Nairn Healthcare Group, Cawdor Rd, Nairn, IV12 5EE UK; 4 NHS Highland, NHS Highland Cardiac Unit Raigmore Hospital, Inverness, IV2 3UJ UK

**Keywords:** COVID-19, Mental health, Psychology, Intervention, Review, Frontline, Staff, Workers, Healthcare, Social care

## Abstract

**Background:**

Health and social care workers (HSCWs) have carried a heavy burden during the COVID-19 crisis and, in the challenge to control the virus, have directly faced its consequences. Supporting their psychological wellbeing continues, therefore, to be a priority. This rapid review was carried out to establish whether there are any identifiable risk factors for adverse mental health outcomes amongst HSCWs during the COVID-19 crisis.

**Methods:**

We undertook a rapid review of the literature following guidelines by the WHO and the Cochrane Collaboration’s recommendations. We searched across 14 databases, executing the search at two different time points. We included published, observational and experimental studies that reported the psychological effects on HSCWs during the COVID-19 pandemic.

**Results:**

The 24 studies included in this review reported data predominantly from China (18 out of 24 included studies) and most sampled urban hospital staff. Our study indicates that COVID-19 has a considerable impact on the psychological wellbeing of front-line hospital staff. Results suggest that nurses may be at higher risk of adverse mental health outcomes during this pandemic, but no studies compare this group with the primary care workforce. Furthermore, no studies investigated the psychological impact of the COVID-19 pandemic on social care staff. Other risk factors identified were underlying organic illness, gender (female), concern about family, fear of infection, lack of personal protective equipment (PPE) and close contact with COVID-19. Systemic support, adequate knowledge and resilience were identified as factors protecting against adverse mental health outcomes.

**Conclusions:**

The evidence to date suggests that female nurses with close contact with COVID-19 patients may have the most to gain from efforts aimed at supporting psychological well-being. However, inconsistencies in findings and a lack of data collected outside of hospital settings, suggest that we should not exclude any groups when addressing psychological well-being in health and social care workers. Whilst psychological interventions aimed at enhancing resilience in the individual may be of benefit, it is evident that to build a resilient workforce, occupational and environmental factors must be addressed. Further research including social care workers and analysis of wider societal structural factors is recommended.

**Supplementary Information:**

The online version contains supplementary material available at 10.1186/s12889-020-10070-3.

## Background

Health and social care workers (HSCWs) continue to play a vital role in our response to the COVID-19 pandemic. It is known that HSCWs exhibit high rates of pre-existing mental health (MH) disorders [1–3] which can negatively impact on the quality of patient care [4].

Studies from previous infectious outbreaks [5, 6] suggest that this group may be at risk of experiencing worsening MH during an outbreak. Current evidence examining the psychological impact on similar groups [7–9], suggest that this group may be at risk of experiencing poor MH as a direct result of the COVID-19 pandemic. Compounding the concerns about these data are that HSCWs will be likely to not only be at a higher risk for experiencing MH problems during the pandemic, but also in its aftermath [5].

There are some specific features of the COVID-19 pandemic that may specifically heighten its potential to impact on the MH of HSCWs.

Firstly, the scale of the pandemic in terms of cases and the number of countries affected has left all with an impression that ‘no-one is safe’. Media reporting of the pandemic has repeatedly focused on the number of deaths in HSCWs and the spread of the disease within health and social care facilities which is likely to have amplified the negative effects on the MH of HSCWs.

Secondly, usual practice has been significantly disrupted and many staff have been asked to work outside of their usual workplace and have been redeployed to higher risk front line jobs.

Finally, the intense focus on personal protective equipment (PPE) is likely to have specifically heightened the impact of COVID-19 on the MH of HSCWs due to the uncertainty surrounding the quantity and quality of equipment, the frequently changing guidance on what PPE is appropriate in specific clinical situations and the uncertainty regarding the absolute risk of transmission posed. While other workers will have been impacted by COVID-19, it is highly likely that the above factors will have disproportionately affected the MH of HSCWs [9, 10]. Indeed a British Medical Association survey on the 14th May 2020 during the pandemic showed that 45% of UK doctors are suffering from depression, anxiety, stress, burnout or other mental health conditions relating to, or made worse by, the COVID-19 crisis [11].

Although evidence based psychological interventions are available for this population [12], there is a paucity of evidence about interventions for the MH of HSCWs during pandemics. Recent calls to action mandated the need to provide high quality data on the psychological impacts of the COVID-19 pandemic [13, 14]. This pandemic has rapidly changed the functioning of society at many levels which suggests that these data are not only needed swiftly, but also with caution and scientific rigour [13, 14].

These data are needed in order to equip HSCWs to do their job effectively – high levels of stress and anxiety have been shown to decrease staff morale, increase absenteeism, lower levels of work satisfaction and quality of care [6, 15]. It is therefore a priority to understand the psychological needs of our HSCWs in order to provide them with the appropriate tools to mitigate the negative effects of dealing with the COVID-19 pandemic.

While HSCWs have been identified as vulnerable to the negative psychological impact from the current pandemic, they do not form a homogeneous population. It may therefore be appropriate to identify particularly vulnerable groups *within* the larger population of HSCWs and target psychological support to them. This review seeks to understand whether any group of HSCWs could be confidently excluded from psychological support interventions because they are deemed to be at a low risk. Holmes et al. [14] have warned that a one-size-fits-all approach to supporting HSCWs might not be effective. This, together with the lack of evidence around tailoring psychological interventions during pandemics [1], highlights the importance of identifying vulnerable groups, to ensure appropriately personalised interventions are made available.

### Aim of the review

The aim of this review is to identify the psychological impact of the COVID-19 pandemic on the health and social care professions, more specifically to identify which sub-groups are most vulnerable to psychological distress and to identify the risk and protective factors associated with this population’s mental health.

This review, looking exclusively at the psychological impact of the COVID-19 pandemic on HSCWs will therefore contribute to informing where mental health interventions, together with organisational and systemic efforts to support this population’s mental health could be focussed in an effort to support psychological well-being [14]. Rapid but robust gathering of evidence to inform health decision-makers is vital and in circumstances such as these, the WHO recommends rapid reviews [16].

## Methods

### Search strategy

Planning, conducting and reporting of this study was based on the guidelines for rapid reviews [17], set by the WHO [16] and the recent COVID-19 Cochrane Collaboration’s recommendations [18].

### Data sources and searches

Two authors (CoM & RP) searched across a broad range of databases to capture research from potentially relevant fields, including health, mental health and health management. Within the OVID platform of databases Medline, EMBase, HMIC and PsychInfo were searched. Within the EbscoHost platform of databases, CINAHL, Medline, APA PsychInfo, Business Source Elite, Health Source and Academic Search Complete were searched. Beyond the OVID and EbscoHost platforms, SCOPUS, the King’s Fund Library, Social Care Online, PROSPERO and Google Advanced were also searched, making 16 databases searched (14 unique databases and two having been searched twice on separate platforms).

Owing to the rapidly changing landscape of the COVID-19 pandemic, and in an effort to include as many eligible papers as possible, the search strategy was executed on 23 April 2020 and again 2 weeks later on 6 May 2020 using a combination of subject headings and keyword searching (see Additional file 1). The bibliographical database was created with EndNote X7™.

### Search criteria

The design of the search criteria was intended to draw together research both for this rapid review, and to contribute to the design of a digital mental health intervention to enhance the psychological well-being of HSCWs. The design of the search criteria is discussed in further detail in the Additional file 1.

### Types of participants

Participants were restricted to HSCWs during the COVID-19 pandemic.

### Types of studies included

Published observational and experimental studies that reported the psychological effects on HSCWs during the COVID-19 pandemic were included. The study designs included quantitative and qualitative primary studies. Studies relating to previous pandemics and epidemics (such as SARS, MERS, H1N1, H5N1, Zika, Ebola, West Nile Fever) were excluded as these results have been reported elsewhere [7]. Reviews, theses, position papers, protocol papers, and studies published in languages other than English were excluded.

### Screening and selection of studies

Searches were screened according to the selection criteria by JDK. The full text of potentially relevant papers was retrieved for closer examination. The reviewer erred on the side of inclusion where there was any doubt, to ensure no potentially relevant papers were missed. The inclusion criteria were then applied against full text versions of the papers (where available) independently by JDK and HL. Disagreements regarding eligibility of studies were resolved by discussion and consensus. Where the two reviewers were still uncertain about inclusion, the other reviewers (RP, CoM) were asked to provide input to reach consensus.

### Data extraction and quality assessment

Relevant data were extracted into structured tables including country, setting, population, study design, number of participants, mental health conditions and their measurement tools and main study results. Where available, we extracted risk factors and protective factors. HL, LE and JDK extracted all the data while JDK checked for accuracy and completeness.

Table 2 presents an overview of the validated tools used per study type to assess study quality and risk of bias. JDK and HL assessed the quality of cross-sectional studies with the Joanna Briggs Institute tool [48] and JDK assessed their risk of bias using the Evidence Partners [49] appraisal tool. JDK assessed the risk of bias for the longitudinal study with the Critical Appraisal Skills Programme (CASP) appraisal tool [50] and the uncontrolled before-after study with the ROBINS – I [51]. SAM utilised Joanna Briggs Institute tool to assess the qualitative studies [38] and the Mixed methods appraisal tool (MMAT) [41] to assess mixed methods studies.

### Data synthesis and analysis

Current best practice guided the tabulated and narrative synthesis of the results [52, 53]. The studies’ outcomes were categorised according to the psychological impact of COVID-19 on HSCWs of:

- general psychological impacts
- the risk factors associated with adverse mental health outcomes
- the protective factors against adverse mental health outcomes

Previous studies’ logical syntheses [6] were adapted by organising the risk and protective factors into psychosocial, occupational, sociodemographic and environmental categories. The GRADE method from the Cochrane Collaboration [54] was used to assess the quality of evidence of outcomes included in this rapid review. Varied study quality, together with study type and outcome heterogeneity precluded performing a meta-analysis.

### Patient and public involvement

Some members of the author team are frontline healthcare staff during the COVID-19 pandemic and contributed to the design of the review.

## Results

### Search results

**Fig. 1:**
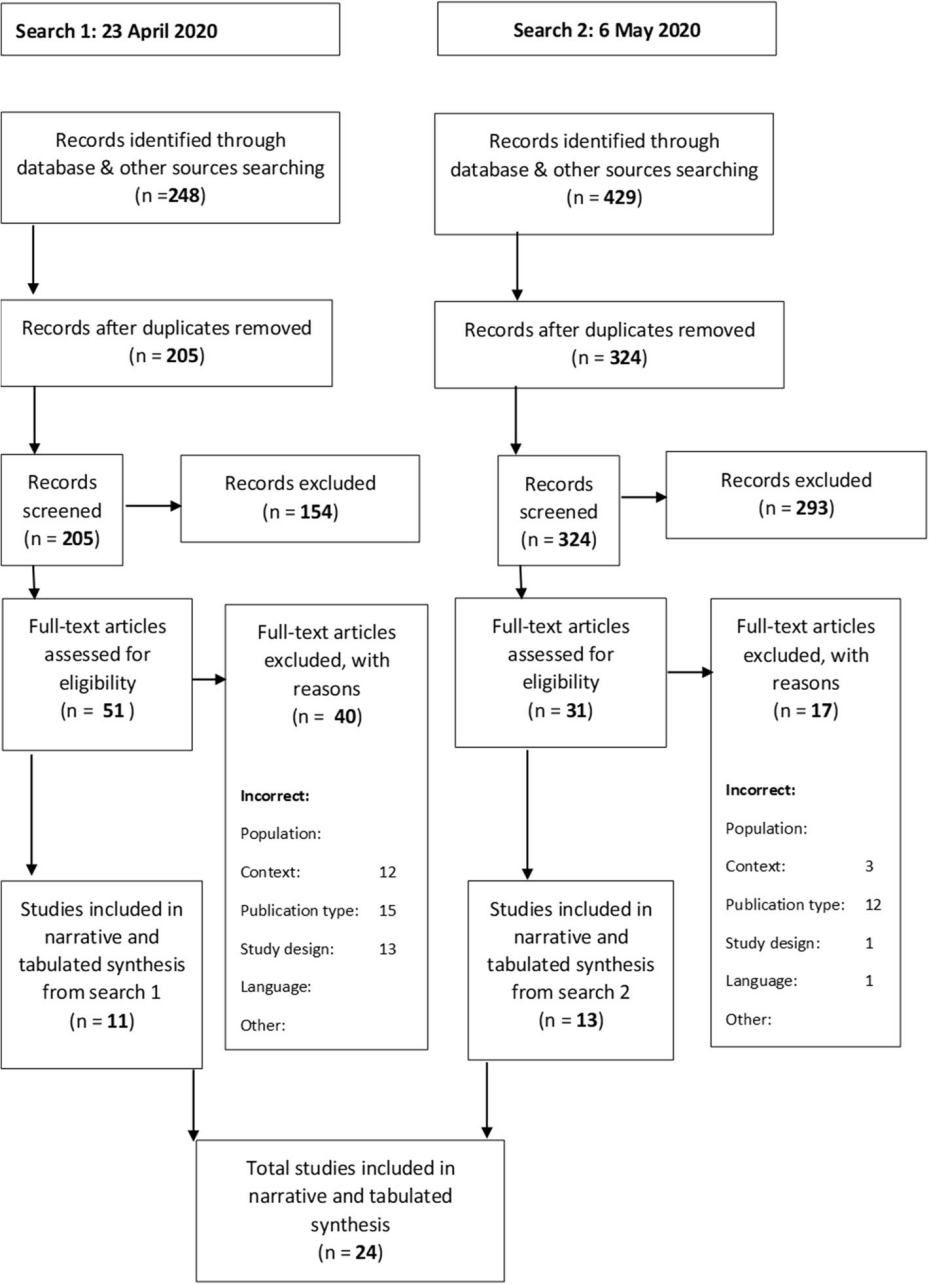
Prisma Flow Diagram

The 677 records of interest were found from the two searches (429 in search 1 and 529 in search 2). After 148 duplicates were removed, 529 records were screened. Of these, 82 full texts of potentially relevant studies were assessed for eligibility (see Fig. 1). Twenty-four published studies met the inclusion criteria for the rapid review.

### Study characteristics

The 24 studies included in this review consisted of 18 cross-sectional, 2 mixed methods, 2 qualitative, 1 longitudinal and 1 uncontrolled before-after study. The total number of participants in these studies was 13,731. In the cross-sectional studies, participant numbers ranged between 59 and 2299. Participant numbers in the two mixed method studies were 37 and 222 respectively, whilst the qualitative studies included 10 and 20 participants, respectively. The longitudinal study included 120 participants and the uncontrolled before-after study, 27 participants. See Table 1 for sampling methods within the included papers. The majority of papers utilised non-probability sampling methods, limiting generalisability of findings. One exception was Lai et al., who used region stratified 2-stage cluster sampling.

**Table 1:** Study Characteristics

	Author	Design	Country	Participants	Selection method	Measures
1	Ahmed et al., (2020) [21]	Cross-sectional	Global (30 countries)	*n* = 650	Online questionnaire distributed via email and social media to dental professionals worldwide.	Validated questionnaire: 22 closed-ended questions divided into two sections. (Fear & Clinical practices)
2	Balakumar et al., (2020) [22]	Uncontrolled before and after study.	UK	*n* = 27 (Surgeons)	Pre- and post-training surveys distributed to a surgical team.	Pre- and post-training surveys
3	H.Cai et al., (2020) [24]	Cross-sectional	China Hunan	*n* = 534 (Frontline medical workers)	Questionnaires sent to frontline medical staff in Hunan province between January and March 2020.	Five-section questionnaire
4	W.Cai et al., (2020) [25]	Cross-sectional	China Jiangsu Province	*n* = 1521 (147 experienced in public health emergencies (PHE))	Health care workers recruited but method unclear.	SCL-90 CD-RISC SSRS
5	Cao et al., (2020) [55]	Mixed methods	China Beijing	*n* = 37 (16 Doctors, 19 Nurses, and 2 Technicians within a COVID-19 clinic)	Qualitative and Quantitative evaluations of staff in a fever clinic. Staff had been handpicked based on their ‘experience, adaptability and tenacity under pressure in past works’	PHQ-9, MBI, Qualitative interviews
6	Chew et al., (2020) [27]	Cross- sectional	Singapore & India	*n* = 906 (480 HCW’s from a Singapore Hospital)	HCWs from 5 major hospitals invited to participated in a questionnaire between Feb 2019 – April 2020.	DASS-21, IES-R Symptom questionnaire
7	Chung & Yeung, (2020) [28]	Cross-sectional	China Hong Kong	*n* = 69 (HCWs: 69/8418 full-time hospital staff)	Online mental health self-assessment questionnaire distributed to all hospital staff in the Hong Kong East Cluster.	PHQ-9
8	Huang & Zhao, (2020) [29]	Cross- sectional	China Nationwide	*n* = 603 (31.1% HCWs)	Web-based survey of general population, invited via social media, random recruitment – all Chinese people using WeChat may have seen it.	Web-based survey. PSQI, GAD, CES-D
9	Kang et al., (2020) [30]	Cross-sectional	China, Wuhan	*n* = 994 (Doctors and Nurses)	Questionnaire distributed online to doctors or nurses working in Wuhan.	PHQ-9, GAD-7, ISI, IES-R
10	Lai et al., (2020) [31]	Cross-sectional	China (Nationwide but 60% from Wuhan)	*n* = 1257 (Nurses and Doctors in 34 hospitals/fever clinics)	Hospital based survey via region-stratified 2-stage cluster sampling from Jan 292,020 – Feb 32,020.	PHQ-9, GAD-7, ISI, IES-R
11	Li et al., (2020) [32]	Cross-sectional	China, Wuhan	*n* = 740 (214 general population and 526 Nurses)	Mobile app-based questionnaire of general public and nurses in Wuhan.	Vicarious Trauma Questionnaire (Chinese version)
12	Liang, Chen, Zheng, & Li, (2020) [56]	Cross-sectional	China, Guangdong Province	*n* = 59 (23 Doctors and 36 Nurses from COVID-19 department and 21 HCWs from other departments)	Questionnaire distributed to medical staff in a hospital. Method of distribution unclear.	SDS, SAS
13	Lu, Wang, Lin & Li, (2020) [34]	Cross-sectional	China, Fujian	*n* = 2299 (2042 Medical and 257 admin staff)	Questionnaire survey of medical staff in a provincial hospital in Feb 2020.	NRS, HAMA, HAMD
14	Mo et al., (2020) [35]	Cross-sectional	China, Wuhan	*n* = 180 (Nurses from Guangxi supporting COVID-19 in Wuhan)	Convenient sampling of nurses from Guangxi recruited to support COVID-19 work in Wuhan. 85.71% response rate of 180 nurses sampled.	SO, SAS
15	Shacham et al., (2020) [36]	Cross- sectional	Israel	*n* = 338 (Dental hygienists and Dentists)	Dental hygienists and dentists, approached using social media, mailing lists and forums.	Demands Scale—Short Version, GSES, Kessler K6
16	Sun et al., (2020) [37]	Qualitative	China, Henan (One hospital)	*n* = 20 (Nurses/17 Female)	Purposeful sampling of nurses caring for COVID-19 patients in a hospital. Jan/Feb 2020.	Semi-structured interviews
17	Tan et al., (2020) [39]	Cross- sectional	Singapore (Two tertiary hospitals)	*n* = 470 (HCWs – medical and non-medical)	HCWs from two major tertiary hospitals in Singapore invited to participate, Feb/March 2020	DASS-21, IES-R
18	Urooj et al., (2020) [40]	Mixed Method	Pakistan	*n* = 222 (134 without COVID-19 patients and 150 female)	Purposive sampling of 250 clinicians from a range of specialities and seniority. 222 responded (88.8%)	Doctors fears and expectations
19	Wang et al., (2020) [57]	Cross-sectional	China, Wuhan	*n* = 123 (HCWs in a Paediatric centre)	Questionnaire survey conducted at a paediatric centre in Wuhan. 50% of all HCWs responded & were included.	PSQI, SAS, SDS
20	Wu et al., (2020) [43]	Cross-sectional	China, Wuhan	*n* = 190 (Hubai cancer hospital – all from oncology 1:1 ratio frontline vs usual wards)	220 physicians and nurses from Hubai cancer hospital invited in March 2020. 190 included.	MBI
21	Xiao et al., (2020) [44]	Cross-sectional	China, Wuhan	n = 180 (54% Nurses and 45.6% Doctors from a respiratory medicine/ fever clinic)	Unclear how participants were sampled. All were medical staff who treated COVID-19 patients in Jan/Feb 2020.	SAS, GSES, SASR, PSQI, SSRS
22	Xu, Xu, Wang & Wang, (2020) [58]	Longitudinal	China, Shanghai	*n* = 120 (Surgical staff. One hospital split into two groups of 60 Grp 1 – Jan-Feb (outbreak period) Grp 2 – March (non-outbreak)	Surgical medical staff sampled.	Anxiety scale, Depression score, Dream anxiety score, SF-36 scale
23	Yin & Zeng, (2020) [46]	Qualitative – in-depth interviews	China, Wuhan	*n* = 10 (Nurses at the front-line; having cared for COVID-19 patients > 1 week)	Purposive sampling	
24	Zhang et al., (2020) [47]	Cross -sectional	China, Nationwide	*n* = 2182 (927 Medical HCWs; 680 Doctors and 247 Nurses, 1255 non-medical HCWs)	Random sampling – anyone in China > 16 years were welcome to join using an online platform.	ISI SCL-90-R PHQ-4 Chinese versions

Measures Description

Depression: CES-D: Centre for Epidemiologic Studies Depression Scale, HAM-D: Hamilton Depression Rating Scale, PHQ-9: Patient Health Questionnaire, SCL-20: Symptom checklist depression scale, SDS: Zung Self-Rating Depression Scale

Anxiety: GAD-7: Generalised Anxiety Disorder Questionnaire, HAM-A: Hamilton Anxiety Rating Scale, SAS: Zung Self-Rating Anxiety Scale

Stress: SOS: Stress Overload Scale

Depression & Anxiety: DASS-21: Depression, Anxiety and Stress Scale, PHQ-4: Patient Health Questionnaire-4

Sleep: PSQI: Pittsburgh Sleep Quality Index, ISI: Insomnia Severity Index

Others: CD-RISC: Connor-Davidson Resilience Scale, Demands Scale—Short Version, Dream anxiety score, GSES: Generalised self-efficacy scale, IES-R: Impact of Event Scale, Kessler K6 Distress Scale, MBI: Maslach Burnout Inventory, SASR: the Stanford Acute Stress Reaction questionnaire, SCL-90: The Symptom Checklist-90-R, SF-36: Short Form Health Survey, SSRS: Social Support Rating Scale, Vicarious Trauma Questionnaire

Eighteen of the studies were from China, of which 8 were based in Wuhan, where the COVID-19 outbreak began. The rest were from America (1), Israel (1), UK (1), Singapore (1), Pakistan (1), multicentre - Singapore & India (1), Global (1). Several validated measures were used to assess anxiety, depression, insomnia, stress and burnout. Table 1 provides an overview of the included studies.

### Risk of bias assessment

The quality of the cross-sectional studies was fair, with 16 studies scoring 6 or higher on the JBI appraisal tool and eleven scoring 7 or higher (a score of 7 and above is an indicator of study quality). The majority of the studies indicated a low risk of bias when assessed with the Evidence Partners’ appraisal tool. The uncontrolled before-after study indicated a high risk of bias. The qualitative studies indicated a good level of quality (JBI scores of 9 & 10 respectively) while mixed methods studies showed varied quality. In the cross sectional studies, the most common problem affecting study quality was failure to deal with confounding factors. Failure to locate the researcher culturally or theoretically affected the qualitative papers, whilst the two mixed methods papers’ study quality was affected by lack of explicitly articulated research questions. A summary of the risk of bias and quality assessments are provided in Table 2.

**Table 2:** Risk of bias and quality assessment summary

	Author	Participants and setting described in detail, including similarity of controls	Criteria for inclusion clearly defined and exposures similarly measured	Exposure measured in valid and reliable way	Objective, standard criteria used for measurement of condition	Confounding factors identified	Strategies to deal with confounding factors stated	Outcomes measured in valid and reliable way	Appropriate statistical analysis used?	JBI Score [19]	Risk of Bias [20]
1	Ahmed et al., 2020 [21]	+	+	+	+	+	–	?	+	6	Low
2	Balakumar et al., 2020 [22]	Risk of bias of uncontrolled before-after studies (assessed with ROBINS – I) [23]: Low quality evidence									
3	H. Cai et al., 2020 [24]	+	+	+	–	+	–	+	+	6	Low
4	W. Cai et al., 2020 [25]	+	+	+	+	+	–	+	+	7	Low
5	Cao et al., 2020 [26]	Mixed methods appraisal tool (MMAT) used (Hong et al., 2018) S1–2 not addressed satisfactory: Low quality evidence									
6	Chew et al., 2020 [27]	+	+	+	+	+	+	+	+	8	Low
7	Chung & Yeung, 2020 [28]	+	–	+	+	–	–	–	–	3	High
8	Huang & Zhao, 2020 [29]	+	+	+	+	+	–	+	+	7	Low
9	Kang et al., 2020 [30]	+	+	+	+	+	+	–	+	7	Low
10	Lai et al., 2020 [31]	+	+	+	+	+	–	+	+	7	Low
11	Li et al., 2020 [32]	+	+	+	+	+	–	+	+	7	Low
12	Liang et al., 2020 [33]	–	–	+	+	–	–	+	+	4	High
13	Lu et al., 2020 [34]	+	+	+	+	+	–	+	+	7	Low
14	Mo et al., 2020 [35]	+	–	+	+	+	–	+	+	6	Minor
15	Shacham et al., 2020 [36]	+	+	+	+	+	–	+	+	7	Low
16	Sun et al., 2020 [37]	Joanna Briggs Institute tool to assess qualitative studies used – 10 item tool [38]: High quality evidence								9	
17	Tan et al., 2020 [39]	+	+	+	+	+	–	+	+	7	Low
18	Urooj et al., 2020 [40]	Mixed methods appraisal tool (MMAT) [41] S1–2 & all 5 criteria addressed satisfactory: High quality evidence									
19	S. Wang et al., 2020 [42]	+	+	+	+	+	–	+	+	7	Low
20	Wu et al., 2020 [43]	+	–	+	+	+	–	+	+	6	Minor
21	Xiao et al., 2020 [44]	+	–	+	+	+	–	+	+	6	Minor
22	Xu et al., 2020 [42]	Assessed with Critical Appraisal Skills Programme appraisal tool [45]									Minor
23	Yin & Zeng, 2020 [46]	Joanna Briggs Institute tool to assess qualitative studies used – 10 item tool [38]: High quality evidence								10	
24	Zhang et al., 2020 [47]	+	+	+	+	+	–	+	+	7	Low

### Psychological toll on healthcare workers

Of the 24 studies included, 22 directly assessed the psychological toll on healthcare workers and all found levels of anxiety, depression, insomnia, distress or Obsessive Compulsive Disorder (OCD) symptoms [24–27, 29–31, 33–37, 39, 40, 42–44, 46, 47, 58–60].

Psychological symptoms were assessed using various validated measures as outlined in Table 3 – the summary of included studies. The most common outcomes assessed were sleep, anxiety and depression. The prevalence of depressive symptoms varied greatly, ranging between 8.9% [39] to 50.4% [31]. These findings suggest marked differences in the prevalence of depressive symptoms across the studies. The prevalence of anxiety in cross-sectional studies ranged between 14.5% [39] to 44.6% [31]. Sleep was also assessed in several studies. Lai et al. [31] found the prevalence of sleep disturbances to be 34%, whilst another, nationwide survey in China found that HCWs had significantly worse sleep than the general population [29].

**Table 3:** Certainty of evidence for the risk factors associated with adverse mental health outcomes on health and care staff during the COVID-19 pandemic

No of studies	Design	Risk of bias	Additional considerations	Certainty (overall score)^**a**^
**Factor: Frontline staff/Close contact with COVID-19 patients** [30, 31, 34, 42]				
4	2	2	Inconsistency: Higher burnout reported in non-frontline staff (Cancer hospital, Wuhan) [43]. Frontline nurses reported lower vicarious trauma scores [32]. No difference between frontline and non-frontline staff reported (This finding was not statistically significant) [33].	**Moderate**
**Factor: Nurse** [24, 26, 31]				
3	2	2	Inconsistency: Doctors were found to have more sleep disturbances than nurses (This finding was not statistically significant) [42]. Not all confounding factors were dealt with in the three studies reporting nurses to be at a higher risk for adverse psychological outcomes. No studies compared nurses to primary care or social staff.	**Moderate**
**Factor: Clinical healthcare workers** [34, 47]				
2	2	2	Inconsistency in these findings [39].	**Moderate**
**Factor: Heavy workload** [26, 35, 37]				
3	2	2	No serious inconsistencies.	**High**
**Factor: Lack of personal protective equipment (PPE)** [24, 28, 40, 46]				
4	2	2	No serious inconsistencies.	**High**
**Factor: Point of outbreak** [37, 58]				
2	2	1	Only two studies - one was limited to the sample of a surgical department where confounding factors were not dealt with and one was a qualitative study.	**Low**
**Factor: Rural location** [47]				
1	2	0	Only one study reported findings on effect of rurality.	**Very low**
**Factor: Fear of infection** [21, 26, 28]				
3	2	2	No serious inconsistencies.	**High**
**Factor: Concern about family** [24, 26, 37, 40]				
4	2	1	This theme was predominantly raised in qualitative literature.	**Moderate**
**Factor: Younger age** [24, 29, 33]				
3	2	2	Age was found to be a complex risk factor where the focus of anxiety depended on the age group assessed [24].	**Low**
**Factor: Gender – Female** [31, 47]				
2	2	1	Inconsistencies were found – for example: a large global survey of dentists found no differences based on gender [21]. Furthermore, confounding factors assessing gender in both included studies were not satisfactorily dealt with.	**Low**
**Factor: Organic illness** [36, 47]				
2	2	1	No serious inconsistencies.	**Moderate**
**Factor: Being an only child** [35, 42]				
2	2	0	No serious inconsistencies.	**Low**

^a^ 4  High = This research provides a very good indication of the likely effect. The likelihood that the effect will be substantially different** is low

3  Moderate = This research provides a good indication of the likely effect. The likelihood that the effect will be substantially different** is moderate

2  Low = This research provides some indication of the likely effect. However, the likelihood that it will be substantially different** is high

1  Very low = This research does not provide a reliable indication of the likely effect. The likelihood that the effect will be substantially different** is very high

** Substantially different = a large enough difference that it might affect a decision

### Risk factors associated with adverse mental health outcomes

Table 3 provides the GRADE evidence profile of the certainty of evidence for the risk factors associated with adverse MH outcomes during the COVID-19 pandemic identified through the review. These risk factors can be grouped into the three thematic areas of i) occupational, ii) psychosocial, iii) environmental.

### Occupational factors

#### Medical HCWs

Two studies showed that medical HCWs (nurses and doctors) had significantly higher levels of MH risk in comparison to non-medical HCWs [34, 47]. Zhang et al. [47] found that medical HCWs had significantly higher levels of insomnia, anxiety, depression, somatization and OCD symptoms in comparison to non-medical HCWs. This was also reflected in a large study in Fujian province, China, in which medical staff had significantly higher anxiety than admin staff [34]. In contrast, Tan et al. [39] found that in a population of 470 HCWs in Singapore, the prevalence of anxiety was significantly higher among non-medical HCWs than medical.

#### Healthcare groups

In three studies nurses were found to be at risk of worse MH outcomes than doctors [24, 26, 31]. One large study in China found nurses were at significant risk of more severe depression and anxiety than doctors [31]. Another found that nurses had significantly higher financial concerns than doctors and felt significantly more anxious on the ward when compared with other groups. There was no significant difference between professionals regarding stopping work or work overload [24]. A mixed method paper also showed that nurses had a higher rate of depressive symptoms than doctors. Whilst this was a small sample size, it echoes the findings from larger studies [26].

With regard to other HCWs, there were two studies which assessed dentists and other dental workers and found them to be at risk of anxiety and elevated distress. Neither study found any difference based on gender or educational level [36, 59]. There were no studies comparing dental workers to other HCWs. We did not find any studies that focussed on the primary care workforce or that assessed social care workers.

With regard to seniority, one paper found that having an intermediate technical title was associated with more severe MH symptoms [31].

#### Frontline staff/direct contact with COVID-19

Four high-quality studies found being in a ‘frontline’ position or having direct contact with COVID-19 patients was associated with higher levels of psychological distress [30, 31, 34, 42].

Increased direct exposure to COVID-19 patients increased the mental health risks in health care workers in one study in Wuhan [30]. This finding is backed by Lai et al. [31], who found that being a frontline worker was independently associated with more severe depression, anxiety and insomnia scores. In addition, a cross sectional survey of staff in a paediatric centre found that contact with COVID-19 patients was independently associated with increased risk of sleep disturbance [42]. Lu et al. [34] found that medical HCWs in direct contact with COVID-19 patients had almost twice the risk of anxiety and depression than non-medical staff with low risk of contact with COVID-19.

There were conflicting results found in two studies. A study in a cancer hospital in Wuhan found burnout frequency to be lower in frontline staff [43]. The authors identified confounding factors which may have led to this result, but it is of interest as it is one of the only studies that assessed HCWs outside of the acute general medicine setting. Li et al. [32], also found that frontline nurses had significantly lower levels of vicarious trauma scores than non-frontline workers and the general population.

#### Personal protective equipment (PPE)

PPE concerns were the most common theme brought up voluntarily in free-text feedback in a study by Chung & Yeung [60], and a survey in Pakistan revealed that 80% of participants expected provision of PPE [40]. H.Cai et al. [24] also found that PPE was protective when adequate, but a risk factor for stress when inadequate. This finding appears to be bolstered by a qualitative study of frontline nurses in Wuhan, which found that physical health and safety was one of their primary needs. This study also reported PPE as a protective factor [46].

#### Heavy workload

Longer working time per week was found to be a risk factor in a study by Mo et al. [35] This, together with increased work intensity or patient load per hour, were themes in a mixed methods study of 37 staff of a clinic in Beijing [26] and a qualitative study of nurses in China [37], also suggesting heavy workload as a risk factor.

### Psychosocial factors

#### Fear of infection

A fear of infection was a highlighted in a qualitative study by Cao et al., (2020, 31), and brought up as a theme in free-text feedback in a cross sectional survey by Chung & Yeung [60]. Ahmed et al. [59] found that 87% of dentists surveyed described a fear of being infected with COVID-19 from either a patient or a co-worker.

#### Concern about family

This was brought up as one of the main stress factors in a study by H.Cai et al. [24], particularly amongst staff in the 31–40 year age-group. Knowing that their family was safe was also the greatest stress reliever [24], whilst fear of infecting family was identified in 79.7% of 222 participants in a study in Pakistan [40]. It was also a theme highlighted in the qualitative data [26, 37].

### Sociodemographic factors

#### Younger age

One Chinese web-based survey which included the general population and HCWs, showed that younger people had significantly higher anxiety and depression scores, but no difference in sleep quality. Conversely, the same study found that HCWs were significantly more likely to have poor sleep quality, but found no difference in anxiety or depressive symptoms based on occupation. The study did not examine the effect of age group on HCWs [29].

H. Cai et al. [24] suggested that age was more complex. They found that all age groups had concerns, but that the focus of their anxieties were different (for example: older staff were more likely to be anxious due to exhaustion from long hours and lack of PPE while younger staff were more likely to worry about their families).

#### Gender

Women were found to be at higher risk for depression, anxiety and insomnia by Lai et al. [31] This was also found to be an independent risk factor for anxiety in another large nationwide Chinese study [47]. However, a global survey of dentists found no differences based on gender [59].

#### Underlying illness

We found two studies which identified that having an underlying organic illness as an independent risk factor for poor psychological outcomes. A study of dentists in Israel found an increase in psychological distress in those with background illnesses as well as an increased fear of contracting COVID-19 and higher subjective overload [36]. In medical HCWs in China, organic illness was found to be an independent risk factor for insomnia, anxiety, OCD, somatising symptoms and depression in medical HCWs [47].

#### Being an only child

This was independently associated with sleep disturbance in paediatric HCWs in Wuhan [42]. Being an only child was also found to be significantly associated with stress by Mo et al. [35].

There was also a significant association between physical symptoms and poor psychological outcomes in a large multicentre study based in India and Singapore. It is unclear if this represented somatization or organic illness and the authors suggest the relationship between physical symptoms and psychological aspects was bi-directional [27].

### Environmental factors

#### Point in pandemic curve

One longitudinal study carried out in China in a surgical department, found that anxiety and depression scores during the ‘outbreak’ period were significantly higher when compared to a similar group assessed after the outbreak period [58]. This was a small sample of 120 and only assessed surgical staff, but this longitudinal data was supported by a qualitative study in China which suggested that anxiety peaks at the start of the outbreak and reduces with time [37].

#### Geography

Living in a rural area was only assessed by one study which showed that it was an independent risk factor for insomnia and anxiety in medical HCWs [47]. This may reflect a need to further investigate the effect of rurality on psychological wellbeing during this pandemic.

### Protective factors against adverse mental health outcomes

The review identified protective factors against adverse mental health outcomes during COVID-19. Table 4 provides the GRADE evidence profile of the certainty of evidence for this. The protective factors can be grouped into the three thematic areas of: i) occupational, ii) psychosocial and iii) environmental.

**Table 4:** Certainty of evidence for the protective factors associated with mitigating adverse mental health outcomes on health and care staff during the COVID-19 pandemic

No of studies	Design	Risk of bias	Additional considerations	Certainty (overall score)^**a**^
**Factor: Support** *(Community, social, team, government)* [24, 37, 44, 46]				
**4**	2	2	No serious inconsistencies.	**High**
**Factor: Adequate personal protective equipment (PPE)** [24, 46]				
**2**	2	0	Few studies assessed PPE directly as a protective factor. Many found it to be a risk factor when inadequate.	**Low**
**Factor: Being in a committed relationship** [36]				
**1**	2	0	Only one study assessed this factor.	**Very low**
**Factor: Prior outbreak experience/COVID-19 Knowledge** [22, 24, 46, 61]				
**4**	2	1	No serious inconsistencies, but the data also included one low quality uncontrolled pre and post exposure study, as well as a qualitative study.	**Moderate**
**Factor: Resilience** [36, 44, 61]				
**3**	2	1	Resilience was empirically measured with validated scores	**High**
**Factor: Altruistic acts** [37]				
**1**	2	1	Only one qualitative study assessed this factor.	**Low**
**Factor: Personal growth** [37]				
**1**	2	1	Only one qualitative study assessed this factor.	**Low**
**Factor: Gratitude, Positive self-attitude** [24, 37]				
**2**	2	1	This factor was not empirically measured.	**Low**
**Factor: Sense of purpose** [37]				
**1**	2	1	Only one qualitative study assessed this factor.	**Low**
**Factor: Safety of family** [24]				
**1**	2	0	Only one study assessed this factor.	**Very low**

^a^4  High = This research provides a very good indication of the likely effect. The likelihood that the effect will be substantially different** is low

3  Moderate = This research provides a good indication of the likely effect. The likelihood that the effect will be substantially different** is moderate

2  Low = This research provides some indication of the likely effect. However, the likelihood that it will be substantially different** is high

1  Very low = This research does not provide a reliable indication of the likely effect. The likelihood that the effect will be substantially different** is very high

** Substantially different = a large enough difference that it might affect a decision

### Occupational factors

#### Experience

W. Cai et al. [25] found that previous experience in a public health emergency (PHE) was protective against adverse mental health outcomes. Staff that had no previous experience were also more likely to have low rates of resilience, and social support.

#### Training

A small cohort study of 27 surgeons, who were given pre and post training surveys, suggested that training alleviates psychological stress [22]. Good hospital guidance was identified to relieve stress in a study by H.Cai et al. [24], and increasing self-knowledge was a coping strategy deployed by staff. Dissemination of knowledge was also mentioned in a qualitative study by Yin & Zeng [46]; participants described subjective stress reduction after their seniors explained relevant knowledge to them.

#### Adequate PPE

As mentioned above, PPE was found to be a protective factor when adequate and a risk factor for poor mental health outcomes when deemed to be inadequate [24, 46].

### Psychosocial factors

#### Resilience

One study assessed self-efficacy in dental staff and found that it was a protective factor [36]. Self-efficacy was also found to improve sleep quality by Xiao et al. [44], whilst W.Cai et al. [25] measured resilience using a validated measure and found it to be a protective factor against adverse MH outcomes.

#### Being in a committed relationship

This was found to be protective by Shacham et al. [36] This was not directly assessed in other studies.

#### Safety of family

This had the biggest impact in reducing stress in a cross-sectional study by H. Cai et al. [24] This was also not assessed in other studies.

### Environmental factors

#### Support

Support and recognition from the health care team, government and community was identified as a protective theme in several studies. Social support, measured using the Social Support Rate Scale (SSRS) was found to indirectly affect sleep by directly reducing anxiety and stress and increasing self-efficacy [44].

Team support was identified as a protective factor in a qualitative study by Sun et al. [37] Good hospital guidance was also identified as a stress reliever by H. Cai et al. [24], who found that HCWs expected recognition from the hospital authorities. This was echoed in a qualitative study of nurses in Wuhan where the desire for community concern was a strong need and tightly linked to the need for PPE and knowledge [46]:

‘*To be honest, I was very apprehensive before coming to the infectious department as support staff, but on the first day here, the head nurse personally explained relevant knowledge such as disinfection and quarantine, and that helped me calm down a lot*.*”*

*“I hope that our society and government pay more attention to lack of personal protective equipment’* [46]*.*

## Discussion

As a communicable disease, and now a global public health emergency (PHE), COVID-19 places a unique challenge on our health and social care workforce that will disrupt not just their usual workplace duties but also their social context [62]. As we adjust to new ways of living and working, HSCWs are likely to continue to face challenges ahead. Our review confirms that the psychological impact of COVID-19 on health care workers is considerable, with significant levels of anxiety, depression, insomnia and distress. Studies revealed a prevalence of depressive symptoms between 8.9–50.4% and anxiety rates between 14.5–44.6% [31, 39]. This is in keeping with other reviews and findings from previous viral outbreaks [7, 8, 63]. The majority of studies published to date come from China, particularly Wuhan - the epicentre of COVID-19. There is minimal evidence published to date on the psychological impact on HCWs in Europe or the US, which have been highly impacted by the pandemic. The studies included in this review were predominantly concerned with hospital settings – we found no studies relating to social care staff or primary care staff. This is a concern, as we have increasing evidence that a large proportion of Western deaths are happening in the community and specifically in care homes [64].

Our review aimed to identify whether there were any groups particularly vulnerable to poor mental health outcomes during COVID-19. We found some evidence that nurses may be at a higher risk than doctors [24, 26, 31]. This is similar to findings which take into account previous viral outbreaks [7]. Confounding factors were not robustly addressed however, and there were no studies that compared nurses with the primary care workforce or social care workers. There was some evidence that clinical HCWs may be at higher risk of psychological distress than non-clinical HCWs [34, 47], but this was not absolute. Tan et al. [39] found a higher prevalence of anxiety among non-medical HCWs in Singapore. The prevalence of poor MH outcomes varied between countries. Chew et al. [27] revealed that in data from India and Singapore, there was an overall lower prevalence of anxiety and depression than similar cross-sectional data from China [27, 31, 39, 60]. This suggests that different contexts and cultures may reveal different findings. It is possible that being at different points in their respective countries’ outbreak curve may have played a part, as there was evidence that this may be influential [58]. Tan et al. [39] postulated that the medical HCWs in Singapore had experienced a SARS outbreak in the past and thus were well prepared for COVID-19 both psychologically and in their infection control measures. What we can deduce is that context and cultural factors are likely to play a role, not just cadre or role of healthcare worker. It also highlights the importance of reviewing the evidence as more data emerges from other countries.

Several risk factors emerged, many in keeping with what has been found in other reviews [7, 8]. Those with the strongest evidence were inadequate PPE [24, 40, 46, 60], fear of infection [26, 59, 60] and heavy workload [26, 35, 37]. Consistent with prior outbreak data [7, 63], there was also good evidence that close contact with COVID-19 cases was a predictor of higher levels of anxiety, depression and insomnia [30, 31, 34, 42], although two studies appeared to show conflicting results [32, 43]. Studies suggested that being younger in age [24, 29, 33] or being female [31, 47, 59] may be a risk factor, however this should be treated with caution. An alternative explanation for this study’s findings may be greater risk of frontline exposure amongst women, who are predominantly employed in lower status roles within healthcare globally according to the WHO [65]. It is important to note that respondents to all studies, when disaggregated by gender, were predominantly female and this may have impacted findings. The consistently higher mortality rate and risk of severe COVID-19 disease amongst men would suggest that the full picture regarding gender and MH during this pandemic is incomplete [66, 67]. Although other risk factors were also identified, their certainty of evidence was deemed to be low.

The majority of cross-sectional studies focussed on measuring adverse MH outcomes which explains the lack of quantitative data on protective factors or coping mechanisms. Of the studies that did assess this, there were protective factors which were associated with adaptive psychological outcomes. Experience of prior infectious disease outbreaks and training were protective against poor mental health outcomes [22, 24, 25, 46]. Adequate PPE was a protective factor when adequate and a risk factor when inadequate [24, 46, 60]. There was good evidence that resilience (measured by self-efficacy or resilience scales) was protective against poor mental health outcomes [25, 36, 44]. This is of importance when assessing how to positively contribute to reducing the psychological burden on our health and social care staff. There was strong evidence that community support was a protective factor [24, 37, 44, 46]. Community support was important in a number of studies, referring to social support as well as recognition and support from the healthcare team, government and wider community [24, 37, 44, 46, 68]. Other adaptive behaviours emerged from qualitative data, including gratitude and the ability to find purpose and growth from the situation [37]. These findings are in keeping with a recent study which identified key domains of risk for burnout in healthcare. They highlighted that being part of a supportive team community is a strong protective factor as are clear values and meaningful work [69]. They advise that organisational-level interventions creating a healthy workplace are the key to preventing burnout [69]. This is echoed in a recent systematic review and meta-analysis of the effectiveness of interventions designed to reduce symptoms and prevalence of MH disorders and suicidal behaviour among physicians. This review concluded that, whilst individually directed interventions are associated with some reduction in symptoms of common MH disorders, there needs to be increased focus on organisational-level interventions that improve the work environment [2].

Whilst our findings showed evidence that occupational and environmental factors at the workplace level played a key role for MH outcomes, there was no mention of wider societal structural issues that have been emerging during this pandemic. Of particular importance is the evidence that black and ethnic minority people of all ages in the global north are at greater risk of contracting and dying from COVID-19 [70–72]. A recent large study in the US found that non-white HCWs were at increased risk of contracting COVID-19 and were disproportionately affected by inadequate PPE and close exposure to COVID-19 patients [3]. This suggests wider structural factors are at play and need to be investigated.

The paucity of empirical studies investigating the mental health of social care and primary care staff during the COVID-19 pandemic should also be rectified. With the majority of studies taking place in China, where ageing in place rather than residential care is the norm [73], it is unsurprising that none investigated care homes, where it is estimated around 40–50% of all deaths related to COVID-19 occur in Europe and the US [64]. Moreover, there is evidence that front-line HCWs who work in nursing homes are among the highest at risk of contracting the virus [3]. With the majority of studies taking place in urban hospital settings, and particularly in Wuhan – the epicentre of the outbreak – the generalizability of findings to other settings may be limited, particularly as countries pass through different points in the outbreak curve. However, this review does highlight the considerable psychological impact that COVID-19 has played so far on health care workers and, therefore, adds to the recent calls to take notice of this important issue [14]. Yet the evidence also suggests that, although predictors for psychological distress exist, these are not absolute and context may play an important role on the manifestation of adverse MH outcomes.

### Strengths and limitations

This rapid review has synthesized and discussed the current literature on the psychological impact of the COVID-19 pandemic on health and social care workers. A major limitation was that no empirical studies investigating this impact on social care workers could be found – limiting generalisability to the population reviewed. Recent evidence also suggests that having an ongoing connection to a paid job, may be protective against poor MH outcomes during the pandemic [74]. It would therefore be useful to compare MH outcomes amongst HCWs, or the general population, who were not actively employed during the pandemic. Unfortunately, none of the studies included this data. Furthermore, job retention schemes have varied widely between countries worldwide, thus limiting the generalisability of findings if this data had been available [75].

However, to our knowledge, this is the first review investigating this population group in the context of COVID-19, without including prior viral outbreaks in its analysis and synthesis. We see this as a strength because this outbreak is different, and worth assessing in its own right. It has affected every country across the globe and disrupted everyday living in a way no other outbreak has in living memory [14]. A major strength of our review is that it endeavoured towards greater inclusion, during the rapidly changing COVID-19 landscape, by completing two runs of the search strategy spaced 2 weeks apart. Whilst we adhered to high methodological standards by assessing study quality and risk of bias, together with using the GRADE approach to evaluate the certainty evidence and following best practice principles [52, 53] to present a narrative and tabulated synthesis, our review remains a rapid one with further clear limitations. The majority of the studies included in this review, for example, were from China and our selection criteria did not include studies from low-income countries or studies in languages other than English - limiting the generalizability of our findings. Being a rapid review, the protocol was not registered on PROSPERO and only one reviewer was responsible for the initial screening of papers and for several of the quality assessments. Finally, as the current review’s searches were carried out early in the pandemic, it will be valuable to consider emerging research from the global arena in the light of this review’s findings.

## Conclusions

This rapid review confirms that front line HCWs are at risk of significant psychological distress as a direct result of the COVID-19 pandemic. Published studies suggest that symptoms of anxiety, depression, insomnia, distress and OCD are found within the healthcare workforce. However, most studies draw only from work in secondary care and none draw from the primary care or social care setting. Published studies so far are predominantly from China (18 out of 24 included studies) and most of these have sampled hospital staff in Wuhan - the epicentre. Findings in this review suggest that the study of different contexts and cultures may reveal different findings and we recommend more research in primary care and social care settings and to monitor rapidly emerging evidence from across the world. This should include analysis of wider societal factors including gender, racial and socio-economic disparities that may influence mental health outcomes in HCWs.

Although risk factors did emerge that were in keeping with evidence from other infectious disease outbreaks, our findings were not absolute. This review suggests that nurses may be at higher risk of adverse MH outcomes during this pandemic, but there were no studies comparing them with social care workers or the primary care workforce. Other risk factors that recurred in the data were heavy workload, lack of PPE, close contact with COVID-19, being female and underlying organic illness. Inconsistencies in findings and lack of data on staff outside hospital settings, suggest that targeting a specific group within health and social care staff with psychological interventions may be misplaced – as both presence of psychological distress and risk factors are spread across the healthcare workforce, rather than associated with particular sub-groups.

A recent call to action for mental health science during COVID-19 recommends research be undertaken to identify interventions that can be delivered under pandemic conditions to mitigate deteriorations in psychological well-being and support mental health. This call to action advised that personalised psychological approaches are likely to be a key [14]. Data from this review suggests that interventions which bolster psychological resilience may be of benefit because this was found to protect against adverse mental health outcomes. Due to the nature of the pandemic which prevents face-to-face interventions, this is likely to be digitally based. A recent systematic review, pre-dating COVID-19, suggested that individualised interventions can have modest effect on reducing adverse mental health outcomes amongst physicians [2]. However, our findings suggest that occupational and environmental factors in the workplace play a key role as risk factors *and* protective factors for mental health outcomes during this pandemic. Heavy workload, proximity to COVID-19 and inadequate PPE were risk factors for poor mental health, whereas good knowledge of COVID-19, a supportive work environment and adequate PPE were protective factors. It would appear from our findings that adequate PPE may be protective not just against infection, but also against adverse mental health outcomes. Individually targeted digital interventions are unlikely to address these factors [2]. We postulate that strengthening psychological resilience in a personalised approach may be effective in protecting our health and social care workers from adverse mental health outcomes but this must not defer responsibility from wider organisations and systems. We suggest that a holistic approach to HCWs psychological wellbeing is needed that includes personalised interventions alongside necessary structural changes to create a healthy, safe and supportive work environment. Further research including social care workers and analysis of wider societal structural factors is recommended.

## Supplementary Information

**Additional file 1.** Search Strategy. This additional file provides a comprehensive overview of the search criteria design as well as the search strategy and pattern.

## Data Availability

The datasets during and/or analysed during the current study available from the corresponding author on reasonable request.

## Abbreviations

CD-RISC: Connor-Davidson Resilience Scale
CES-D: Centre for Epidemiologic Studies Depression Scale (CES-D)
COVID-19: Coronavirus disease 2019
DASS-21: Depression, Anxiety and Stress Scale
GAD-7: Generalised Anxiety Disorder Questionnaire
GRADE: The Grades of Recommendation, Assessment, Development and Evaluation Working Group
GSES: Generalised self-efficacy scale
HAM-A: Hamilton Anxiety Rating Scale
HAM-D: Hamilton Depression Rating Scale
HCWs: Healthcare workers
HSCWs: Health and social care workers
IES-R: Impact of Event Scale
ISI: Insomnia Severity Index
MBI: Maslach Burnout Inventory (MBI)
MH: Mental health
PHE: Public Health Emergency
PHQ-4: Patient Health Questionnaire-4
PHQ-9: Patient Health Questionnaire
PPE: Personal protective equipment
PSQI: Pittsburgh Sleep Quality Index
SAS: Zung Self-Rating Anxiety Scale
SASR: The Stanford Acute Stress Reaction questionnaire
SCL-20: Symptom checklist depression scale
SCL-90: The Symptom Checklist-90-R
SDS: Zung Self-Rating Depression Scale
SF-36: Short Form Health Survey (SF-36)
SOS: Stress Overload Scale
SSRS: Social Support Rating Scale
WHO: World Health Organisation
