# Supplementary material for: A rapid review of the impact of COVID-19 on the mental health of healthcare workers: implications for supporting psychological well-being
Source: BMC Public Health. 2021 Jan 9;21:104. doi: 10.1186/s12889-020-10070-3 (PMC7794640; doi:10.1186/s12889-020-10070-3)

# **Additional file 1** Search criteria design

the Relevant concepts relating to the search criteria were:

- The virus itself
- The virus being classified as a pandemic
- Health and social care workers working in close proximity to the virus, and therefore exposed to the most immediate and consistent heightened risk (based on the findings from previous viral outbreaks)
- Health and social care workers not working in close proximity to the virus, but perhaps still at heightened risk (based on the findings from previous viral outbreaks)
- The mental health impact to this population of the heightened exposure to this risk

Both COVID-19 and any published or disseminated research is new and emerging, therefore, there was no pre-requisite for setting a date range criterion within the search parameters. However, this is not the first strain of a coronavirus or other potentially relevant viral outbreak to occur. In order to delineate content specific to COVID-19, potential variant outbreaks of disease such as SARS, Ebola, H1N1, Middle East respiratory syndrome and equine influenza were not included.
To cover the aspect of COVID-19’s efficacy as a sudden acute respiratory syndrome the term SARS is utilised, however, this is constructed so as to exclude SARS itself as a defined strain of virus with equivalent nomenclature. Research literature on the virus outbreaks highlighted above were excluded herein due to i). time and scope constraints in examining the literature for the purposes of a rapid review; ii). they are epidemics, rather than pandemics, meaning; iii). the inherent scale as well as isolated geographical variations of these outbreaks made examination of such evidence less specific to the focus of this review, and that; iv). reviewers would need to decipher relevant literature to delineate local variables, with inherent risks of diffusion of the focus of the review.

The design of the literature search aimed to find scientific research on which to assess both the psychological needs as well as potential psychological distress and protective factors of the population group: Health and Social Care workers.

**Search Pattern**

**For the sake of methodology the exact same search pattern was replicated for Search 2 (6 May 2020) from Search 1 (23 April 2020)**

**Ovid MEDLINE(R) ALL <1946 to April 23, 2020>**

| Search history sorted by search number ascending | | | |  |  |  |
| --- | --- | --- | --- | --- | --- | --- |
| **#** | **Searches** | **Results** | **Type** |  |  |  |
|  | | | | | | |
| 1 | (severe acute respiratory syndrome coronavirus 2 or Wuhan coronavirus* or 2019-nCoV or 2019ncov or cov-2 or cov2 or Wuhan seafood market pneumonia virus or COVID-19-2019 or COVID-192019 or coronavirus disease 2019 or SARS-CoV-2 or sarscov2 or SARS2 or COVID-19 or COVID-19-19 or 2019 novel coronavirus infection* or coronavirus disease-19 or novel coronavirus or SARS-CoV-2019 or sars-COVID-19 or sars-COVID-192019 or sars-COVID-19 2019 or wuhan virus or wn-cov or wncov or wuhan novel or wnCOVID-19 or wn-COVID-19 or wncov19 or wnCOVID-19 19 or wncov2019).af. | 7154 | Advanced |  |  |  |
| 2 | exp Mental Health/ | 37290 | Advanced |  |  |  |
| 3 | 1 and 2 | 34 | Advanced |  |  |  |
| 4 | exp Health Personnel/ | 507570 | Advanced |  |  |  |
| 5 | 3 and 4 | 11 | Advanced |  |  |  |
| 6 | (mental distress or mental stress*).af. | 5081 | Advanced |  |  |  |
| 7 | 1 and 6 | 0 | Advanced |  |  |  |
| 8 | exp stress/ or exp emotions/ | 237296 | Advanced |  |  |  |
| 9 | 1 and 4 and 8 | 5 | Advanced |  |  |  |
| 10 | 5 or 9 | 13 | Advanced |  |  |  |
| 11 | 7 or 9 or 10 | 13 | Advanced |  |  |  |
| 12 | (mental health or stress*).af. | 1251957 | Advanced |  |  |  |
| 13 | 1 or 2 or 6 or 12 | 1259372 | Advanced |  |  |  |
| 14 | (health personnel or health* worker*).af. | 196570 | Advanced |  |  |  |
| 15 | 13 and 14 | 18342 | Advanced |  |  |  |
| 16 | 1 and 15 | 209 | Advanced |  |  |  |
| 17 | 11 or 16 | 212 | Advanced |  |  |  |
| 18 | exp Residential Facilities/ or exp Homes for the Aged/ or exp Nursing Homes/ | 51940 | Advanced |  |  |  |
| 19 | exp Caregivers/ | 35752 | Advanced |  |  |  |
| 20 | (carer* or caregiver* or care giver*).af. | 88195 | Advanced |  |  |  |
| 21 | 18 and 19 and 20 | 1420 | Advanced |  |  |  |
| 22 | 1 and 2 and 21 | 0 | Advanced |  |  |  |
| 23 | 1 and 21 | 0 | Advanced |  |  |  |
|  | | |  |  | | |

**Embase <1974 to 2020 April 23>**

| Search history sorted by search number ascending | | | |  |  |  |
| --- | --- | --- | --- | --- | --- | --- |
| **#** | **Searches** | **Results** | **Type** |  |  |  |
|  | | | | | | |
| 1 | (severe acute respiratory syndrome coronavirus 2 or Wuhan coronavirus* or 2019-nCoV or 2019ncov or cov-2 or cov2 or Wuhan seafood market pneumonia virus or COVID-19-2019 or COVID-192019 or coronavirus disease 2019 or SARS-CoV-2 or sarscov2 or SARS2 or COVID-19 or COVID-19-19 or 2019 novel coronavirus infection* or coronavirus disease-19 or novel coronavirus or SARS-CoV-2019 or sars-COVID-19 or sars-COVID-192019 or sars-COVID-19 2019 or wuhan virus or wn-cov or wncov or wuhan novel or wnCOVID-19 or wn-COVID-19 or wncov19 or wnCOVID-19 19 or wncov2019).af.  . | 5513 | Advanced |  |  |  |
| 2 | exp Mental Health/ | 152913 | Advanced |  |  |  |
| 3 | 1 and 2 | 91 | Advanced |  |  |  |
| 4 | exp Health Personnel/ | 1517122 | Advanced |  |  |  |
| 5 | 3 and 4 | 30 | Advanced |  |  |  |
| 6 | (mental distress or mental stress*).af. | 83314 | Advanced |  |  |  |
| 7 | 1 and 6 | 26 | Advanced |  |  |  |
| 8 | exp stress/ or exp emotions/ | 815406 | Advanced |  |  |  |
| 9 | 1 and 4 and 8 | 57 | Advanced |  |  |  |
| 10 | 5 or 9 | 76 | Advanced |  |  |  |
| 11 | 7 or 9 or 10 | 85 | Advanced |  |  |  |
| 12 | (mental health or stress*).af. | 1719665 | Advanced |  |  |  |
| 13 | 1 or 2 or 6 or 12 | 1735475 | Advanced |  |  |  |
| 14 | (health personnel or health* worker*).af. | 117558 | Advanced |  |  |  |
| 15 | 13 and 14 | 11326 | Advanced |  |  |  |
| 16 | 1 and 15 | 132 | Advanced |  |  |  |
| 17 | 11 or 16 | 206 | Advanced |  |  |  |
| 18 | exp Residential Facilities/ or exp Homes for the Aged/ or exp Nursing Homes/ | 61972 | Advanced |  |  |  |
| 19 | exp Caregivers/ | 79082 | Advanced |  |  |  |
| 20 | (carer* or caregiver* or care giver*).af. | 130620 | Advanced |  |  |  |
| 21 | 18 and 19 and 20 | 2556 | Advanced |  |  |  |
| 22 | 1 and 2 and 21 | 0 | Advanced |  |  |  |
| 23 | 1 and 21 | 0 | Advanced |  |  |  |

Top of Form

| **APA PsycInfo <2002 to April Week 2 2020>**   \| Search history sorted by search number ascending \| \| \| \| \| --- \| --- \| --- \| --- \| \| **#** \| **Searches** \| **Results** \| **Type** \| \|  \| \| \| \| \| \| \| \| 1 \| (severe acute respiratory syndrome coronavirus 2 or Wuhan coronavirus* or 2019-nCoV or 2019ncov or cov-2 or cov2 or Wuhan seafood market pneumonia virus or COVID-19-2019 or COVID-192019 or coronavirus disease 2019 or SARS-CoV-2 or sarscov2 or SARS2 or COVID-19 or COVID-19-19 or 2019 novel coronavirus infection* or coronavirus disease-19 or novel coronavirus or SARS-CoV-2019 or sars-COVID-19 or sars-COVID-192019 or sars-COVID-19 2019 or wuhan virus or wn-cov or wncov or wuhan novel or wnCOVID-19 or wn-COVID-19 or wncov19 or wnCOVID-19 19 or wncov2019).af. \| 44 \| Advanced \|  \|  \|  \| \| 2 \| exp Mental Health/ \| 52393 \| Advanced \|  \|  \|  \| \| 3 \| 1 and 2 \| 5 \| Advanced \|  \|  \|  \| \| 4 \| exp Health Personnel/ \| 110308 \| Advanced \|  \|  \|  \| \| 5 \| 3 and 4 \| 2 \| Advanced \|  \|  \|  \| \| 6 \| (mental distress or mental stress*).af. \| 11330 \| Advanced \|  \|  \|  \| \| 7 \| 1 and 6 \| 1 \| Advanced \|  \|  \|  \| \| 8 \| exp stress/ or exp emotions/ \| 282480 \| Advanced \|  \|  \|  \| \| 9 \| 1 and 4 and 8 \| 2 \| Advanced \|  \|  \|  \| \| 10 \| 5 or 9 \| 3 \| Advanced \|  \|  \|  \| \| 11 \| 7 or 9 or 10 \| 4 \| Advanced \|  \|  \|  \| \| 12 \| (mental health or stress*).af. \| 880952 \| Advanced \|  \|  \|  \| \| 13 \| 1 or 2 or 6 or 12 \| 881803 \| Advanced \|  \|  \|  \| \| 14 \| (health personnel or health* worker*).af. \| 49142 \| Advanced \|  \|  \|  \| \| 15 \| 13 and 14 \| 25848 \| Advanced \|  \|  \|  \| \| 16 \| 1 and 15 \| 11 \| Advanced \|  \|  \|  \| \| 17 \| 11 or 16 \| 13 \| Advanced \|  \|  \|  \| \| 18 \| exp Residential Facilities/ or exp Homes for the Aged/ or exp Nursing Homes/ \| 5294 \| Advanced \|  \|  \|  \| \| 19 \| exp Caregivers/ \| 22438 \| Advanced \|  \|  \|  \| \| 20 \| (carer* or caregiver* or care giver*).af. \| 120713 \| Advanced \|  \|  \|  \| \| 21 \| 18 and 19 and 20 \| 368 \| Advanced \|  \|  \|  \| \| 22 \| 1 and 2 and 21 \| 0 \| Advanced \|  \|  \|  \| \| 23 \| 1 and 21 \| 0 \| Advanced \|  \|  \|  \| \| 24 \| 1 and 21 \| 0 \| Advanced \|  \|  \|  \| \| 25 \| exp carers/ \| 0 \| Advanced \|  \|  \|  \| \| 26 \| 1 and 25 \| 0 \| Advanced \|  \|  \|  \| \|  \| \| \|  \|  \| \| \| |
| --- | --- | --- | --- | --- | --- | --- | --- | --- | --- | --- | --- | --- | --- | --- | --- | --- | --- | --- | --- | --- | --- | --- | --- | --- | --- | --- | --- | --- | --- | --- | --- | --- | --- | --- | --- | --- | --- | --- | --- | --- | --- | --- | --- | --- | --- | --- | --- | --- | --- | --- | --- | --- | --- | --- | --- | --- | --- | --- | --- | --- | --- | --- | --- | --- | --- | --- | --- | --- | --- | --- | --- | --- | --- | --- | --- | --- | --- | --- | --- | --- | --- | --- | --- | --- | --- | --- | --- | --- | --- | --- | --- | --- | --- | --- | --- | --- | --- | --- | --- | --- | --- | --- | --- | --- | --- | --- | --- | --- | --- | --- | --- | --- | --- | --- | --- | --- | --- | --- | --- | --- | --- | --- | --- | --- | --- | --- | --- | --- | --- | --- | --- | --- | --- | --- | --- | --- | --- | --- | --- | --- | --- | --- | --- | --- | --- | --- | --- | --- | --- | --- | --- | --- | --- | --- | --- | --- | --- | --- | --- | --- | --- | --- | --- | --- | --- | --- | --- | --- | --- | --- | --- | --- | --- | --- | --- | --- | --- | --- | --- | --- | --- | --- | --- | --- | --- | --- | --- | --- | --- | --- | --- | --- | --- | --- | --- | --- | --- | --- | --- | --- | --- | --- | --- | --- |

Bottom of Form

**HMIC Health Management Information Consortium <1979 to March 2020>**

| Search history sorted by search number ascending | | | |  |  |  |
| --- | --- | --- | --- | --- | --- | --- |
| **#** | **Searches** | **Results** | **Type** |  |  |  |
|  | | | | | | |
| 1 | (severe acute respiratory syndrome coronavirus 2 or Wuhan coronavirus* or 2019-nCoV or 2019ncov or cov-2 or cov2 or Wuhan seafood market pneumonia virus or COVID-19-2019 or COVID-192019 or coronavirus disease 2019 or SARS-CoV-2 or sarscov2 or SARS2 or COVID-19 or COVID-19-19 or 2019 novel coronavirus infection* or coronavirus disease-19 or novel coronavirus or SARS-CoV-2019 or sars-COVID-19 or sars-COVID-192019 or sars-COVID-19 2019 or wuhan virus or wn-cov or wncov or wuhan novel or wnCOVID-19 or wn-COVID-19 or wncov19 or wnCOVID-19 19 or wncov2019).af. | 13 | Advanced |  |  |  |
| 2 | exp Mental Health/ | 6354 | Advanced |  |  |  |
| 3 | 1 and 2 | 0 | Advanced |  |  |  |
| 4 | exp Health Personnel/ | 0 | Advanced |  |  |  |
| 5 | 3 and 4 | 0 | Advanced |  |  |  |
| 6 | (mental distress or mental stress*).af. | 143 | Advanced |  |  |  |
| 7 | 1 and 6 | 0 | Advanced |  |  |  |
| 8 | exp stress/ or exp emotions/ | 7152 | Advanced |  |  |  |
| 9 | 1 and 4 and 8 | 0 | Advanced |  |  |  |
| 10 | 5 or 9 | 0 | Advanced |  |  |  |
| 11 | 7 or 9 or 10 | 0 | Advanced |  |  |  |
| 12 | (mental health or stress*).af. | 31832 | Advanced |  |  |  |
| 13 | 1 or 2 or 6 or 12 | 31869 | Advanced |  |  |  |
| 14 | (health personnel or health* worker*).af. | 1764 | Advanced |  |  |  |
| 15 | 13 and 14 | 356 | Advanced |  |  |  |
| 16 | 1 and 15 | 4 | Advanced |  |  |  |
| 17 | 11 or 16 | 4 | Advanced |  |  |  |
| 18 | exp Residential Facilities/ or exp Homes for the Aged/ or exp Nursing Homes/ | 1699 | Advanced |  |  |  |
| 19 | exp Caregivers/ | 0 | Advanced |  |  |  |
| 20 | (carer* or caregiver* or care giver*).af. | 9781 | Advanced |  |  |  |
| 21 | 18 and 19 and 20 | 0 | Advanced |  |  |  |
| 22 | 1 and 2 and 21 | 0 | Advanced |  |  |  |
| 23 | 1 and 21 | 0 | Advanced |  |  |  |
| 24 | 1 and 21 | 0 | Advanced |  |  |  |
| 25 | exp carers/ | 4322 | Advanced |  |  |  |
| 26 | 1 and 25 | 0 | Advanced |  |  |  |
|  | | |  |  |  |  |

**CINAHL**

| **Search ID#** | **Search Terms** | **Search Options** | **Last Run Via** | **Results** |
| --- | --- | --- | --- | --- |
| S14 | S1 AND S12 | Search modes - Boolean/Phrase | Interface - EBSCOhost Research Databases  Search Screen - Basic Search  Database - CINAHL | 42 |
| S13 | S1 AND S11 AND S12 | Search modes - Boolean/Phrase | Interface - EBSCOhost Research Databases  Search Screen - Basic Search  Database - CINAHL | 3 |
| S12 | S4 OR S5 OR S6 OR S7 OR S8 OR S9 OR S10 | Search modes - Boolean/Phrase | Interface - EBSCOhost Research Databases  Search Screen - Basic Search  Database - CINAHL | 215,113 |
| S11 | S2 OR S3 | Search modes - Boolean/Phrase | Interface - EBSCOhost Research Databases  Search Screen - Basic Search  Database - CINAHL | 626,169 |
| S10 | carer or caRERS OR CAREGIVER* OR CARE GIVER* | Search modes - Boolean/Phrase | Interface - EBSCOhost Research Databases  Search Screen - Basic Search  Database - CINAHL | 83,331 |
| S9 | (MH "Residential Facilities+") | Search modes - Boolean/Phrase | Interface - EBSCOhost Research Databases  Search Screen - Basic Search  Database - CINAHL | 33,592 |
| S8 | (MH "Residential Care+") | Search modes - Boolean/Phrase | Interface - EBSCOhost Research Databases  Search Screen - Basic Search  Database - CINAHL | 8,515 |
| S7 | (MH "Caregivers") | Search modes - Boolean/Phrase | Interface - EBSCOhost Research Databases  Search Screen - Basic Search  Database - CINAHL | 37,452 |
| S6 | (MH "Occupational Health+") OR (MH "Occupational Health Services+") | Search modes - Boolean/Phrase | Interface - EBSCOhost Research Databases  Search Screen - Basic Search  Database - CINAHL | 70,596 |
| S5 | (MH "Work Environment+") | Search modes - Boolean/Phrase | Interface - EBSCOhost Research Databases  Search Screen - Basic Search  Database - CINAHL | 35,120 |
| S4 | (MH "Stress, Occupational+") | Search modes - Boolean/Phrase | Interface - EBSCOhost Research Databases  Search Screen - Basic Search  Database - CINAHL | 27,458 |
| S3 | (MH "Mental Disorders+") | Search modes - Boolean/Phrase | Interface - EBSCOhost Research Databases  Search Screen - Basic Search  Database - CINAHL | 602,230 |
| S2 | (MH "Mental Health") | Search modes - Boolean/Phrase | Interface - EBSCOhost Research Databases  Search Screen - Basic Search  Database - CINAHL | 37,989 |
| S1 | (severe acute respiratory syndrome coronavirus 2 or Wuhan coronavirus* or 2019-nCoV or 2019ncov or cov-2 or cov2 or Wuhan seafood market pneumonia virus or COVID-19-2019 or COVID-192019 or coronavirus disease 2019 or SARS-CoV-2 or sarscov2 or SARS2 or COVID-19 or COVID-19-19 or 2019 novel coronavirus infection* or coronavirus disease-19 or novel coronavirus or SARS-CoV-2019 or sars-COVID-19 or sars-COVID-192019 or sars-COVID-19 2019 or wuhan virus or wn-cov or wncov or wuhan novel or wnCOVID-19 or wn-COVID-19 or wncov19 or wnCOVID-19 19 or wncov2019).af. | Search modes - Boolean/Phrase | Interface - EBSCOhost Research Databases  Search Screen - Basic Search  Database - CINAHL | 1,228 |

**Kings Fund Library/Social Care Online**

Variations on textword – eg COVID-19* And Carer* and COVID-19* and vulnerable

**Prospero**

"2019 nCoV" OR 2019nCoV OR "2019 novel coronavirus" OR "COVID-19 19" OR COVID-19 OR "new coronavirus" OR "novel coronavirus" OR "SARS CoV-2" OR (Wuhan AND coronavirus) OR "COVID-19 19" OR "SARS-CoV" OR "2019-nCoV" OR "SARS-CoV-2" and (carer* or caregiver* or "care giver*" or mental) NOT Animal:DB

**SCOPUS**

(severe acute respiratory syndrome coronavirus 2 or Wuhan coronavirus* or 2019-nCoV or 2019ncov or cov-2 or cov2 or Wuhan seafood market pneumonia virus or COVID-19-2019 or COVID-192019 or coronavirus disease 2019 or SARS-CoV-2 or sarscov2 or SARS2 or COVID-19 or COVID-19-19 or 2019 novel coronavirus infection* or coronavirus disease-19 or novel coronavirus or SARS-CoV-2019 or sars-COVID-19 or sars-COVID-192019 or sars-COVID-19 2019 or wuhan virus or wn-cov or wncov or wuhan novel or wnCOVID-19 or wn-COVID-19 or wncov19 or wnCOVID-19 19 or wncov2019).af. AND ( TITLE-ABS-KEY ( stress* OR mental OR carer OR carers OR caregiver OR "care-givers" ) )

**Google Advanced**

Various keyword searches eg COVID-19 and vulnerable, COVID-19 and mental, using no domain, and also using the domains .nhs.uk, ac.uk and org.uk.


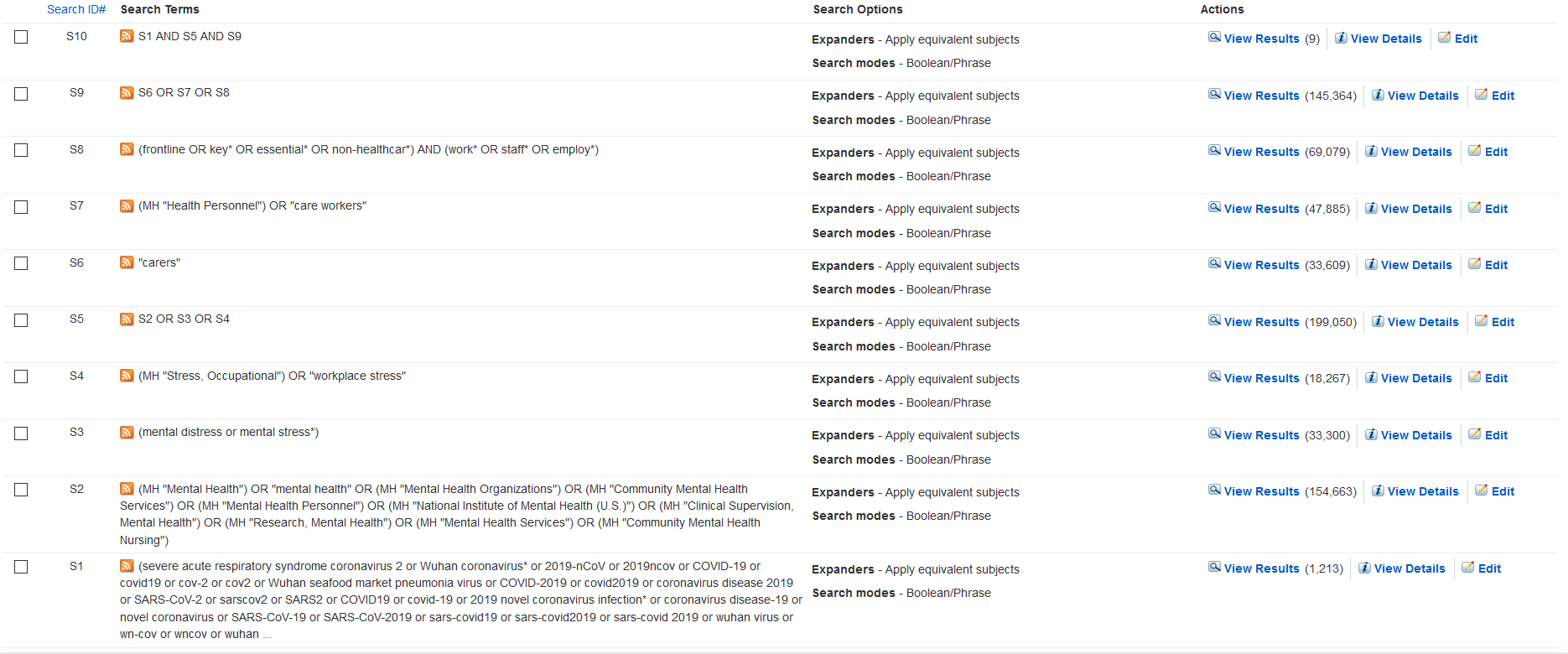

Supplement: Supplementary file 1 — Additional file 1. Search Strategy. This additional file provides a comprehensive overview of the search criteria design as well as the search strategy and pattern. [file 12889_2020_10070_MOESM1_ESM.docx]
